# Significant association between systemic inflammation response index and prognosis in patients with urological malignancies

**DOI:** 10.3389/fimmu.2025.1518647

**Published:** 2025-02-26

**Authors:** Wangbin Ma, Rongqiang Liu, Xinyi Li, Jia Yu, Weixing Wang

**Affiliations:** ^1^ Department of General Surgery, Renmin Hospital of Wuhan University, Wuhan, China; ^2^ Laboratory of General Surgery, Renmin Hospital of Wuhan University, Wuhan, China; ^3^ Hubei Key Laboratory of Digestive System Disease, Wuhan, China

**Keywords:** systemic inflammation response index, urological malignancies, biomarker, prognosis, meta-analysis

## Abstract

**Background:**

The systemic inflammation response index (SIRI) as an immune marker, is associated with prognosis of urological malignancies(UM). However, the conclusion remains controversial. Therefore, the objective of this study was to conduct a meta-analysis to comprehensively evaluate the predictive value of SIRI in patients with UM.

**Methods:**

A comprehensive search of PubMed, Web of Science, and EMBASE databases was performed for articles investigating the association between SIRI and UM. The search deadline was August 28, 2024. Survival outcome such as overall survival (OS), disease-free survival (DFS), progression-free survival (PFS), and recurrence-free survival (RFS) were analyzed.

**Results:**

15 studies from 13 articles involving 4985 patients were included in the meta-analysis. The results showed that increased SIRI was associated with poorer OS (HR: 2.16, 95% CI: 1.61-2.89) and DFS/PFS/RFS (HR: 3.56, 95% CI: 1.41-8.99). Subgroup analysis further confirmed the prognostic value of SIRI in urinary system cancer.

## Introduction

Urological malignancies(UM) has become a great threat to human health. Renal cell carcinoma, bladder cancer, and prostate cancer rank among the most common types of UM, together representing about 12.3% of cancer cases and 7.7% of cancer-related deaths (1). It is expected that incidence and mortality of urinary system cancer would continue to rise worldwide (2). Even with considerable progress in surgical techniques and medical interventions such as chemotherapy, radiotherapy and immunotherapy, the five-year survival rate of UM patients remains alarmingly low (3, 4). Therefore, there is an urgent requirement to identify novel biomarkers prognostic markers for UM.

Inflammation and immune status play important roles in tumor biology (5, 6). Inflammatory response promotes cell proliferation by upregulating cytokines and producing inflammatory mediators (7). Inflammation can also lead to malnutrition, leading to a poor prognosis for cancer patients (8). Inflammation can also impair immune cell function, leading to immunosuppression (9). Immune system restrained tumor cell proliferation, angiogenesis and metastasis through by down-regulating the production of cytokines and inflammatory mediators (10). Many blood indicators, such as neutrophil to lymphocyte ratio, platelet to lymphocyte ratio and albumin-globulin ratio, have been confirmed to predict the prognosis of patients with UM (11–13). However, due to limitations, they were not widely used.

Systemic inflammation response index(SIRI) as a prognostic marker, was first proposed by Qi et al. in 2016 and was composed of neutrophil x monocyte)/lymphocyte count (14). The SIRI score was widely used as a screening tool to assess inflammation and immune status in cancer patients. Many studies have confirmed that SIRI can be used to evaluate patient prognosis in different cancers including non-small cell lung cancer, gastric cancer, breast cancer, ovarian cancer and hepatocellular carcinoma (15–19). Compared with neutrophil to lymphocyte ratio (NLR) and platelet to lymphocyte ratio (PLR), SIRI had higher prediction accuracy (20, 21). The SIRI incorporates three different cells, and may better reflect the inflammatory and immune status of tumor patients. In UM, some studies found that SIRI was associated with prognosis (22, 23). However, the results were still disputed. Whether SIRI was a reliable prognostic marker for UM was unclear. Therefore, we conducted the meta-analysis to summarize the prognostic significance of SIRI in UM.

## Methods

### Literature search strategies

A comprehensive search of PubMed, Web of Science, and EMBASE databases was performed for articles investigating the association between SIRI and UM. The search deadline was August 28, 2024. The following search terms were used: “systemic inflammation response index” OR “system inflammation response index” OR “systemic inflammatory response index” AND cancer OR tumor OR carcinoma OR neoplasm OR malignancy OR tumor AND prognosis OR prognostic OR survival OR outcome. There were no language restrictions. The references for the selected studies were carefully checked for possible studies. This analysis followed the PRISMA guidelines(**Supplementary Material**).

### Study selection criteria

The inclusion: (1) study evaluated the association between SIRI and survival outcomes in patients with UM. (2)provided adequate data to compute the hazard ratios (HRs) with its 95% confidence intervals (CIs). Exclusion criteria: (1) study with insufficient data for calculating HRs and 95% CIs. (2) abstracts, case reports, reviews or letters.

### Data extraction and quality evaluation

The process of extracting data was independently conducted by two researchers, and any differences were settled by discussions. The gathered data included the lead author’s name, publication year, treatment method, cancer type, analytical method and survival outcomes. The Newcastle-Ottawa Scale (NOS) was employed to assess the quality of the incorporated studies (24).

### Statistical analysis

Meta-analysis was performed using STATA software. The aggregated data was evaluated by calculating HRs and 95% CIs. When I^2^ was less than 50%, a fixed effect model was used, and when I^2^ was more than 50%, a random effect model was used. Subgroup analysis was analyzed to further explore the predictive significance of SIRI in UM. The strength of the findings was assessed using sensitivity analysis. Begg’s test, Egger’s test, and the trim-and-fill methods were used to investigate publication bias (25–27).The p value less than 0.05 was considered statistically significant.

## Results

### Search results

**Figure 1:**
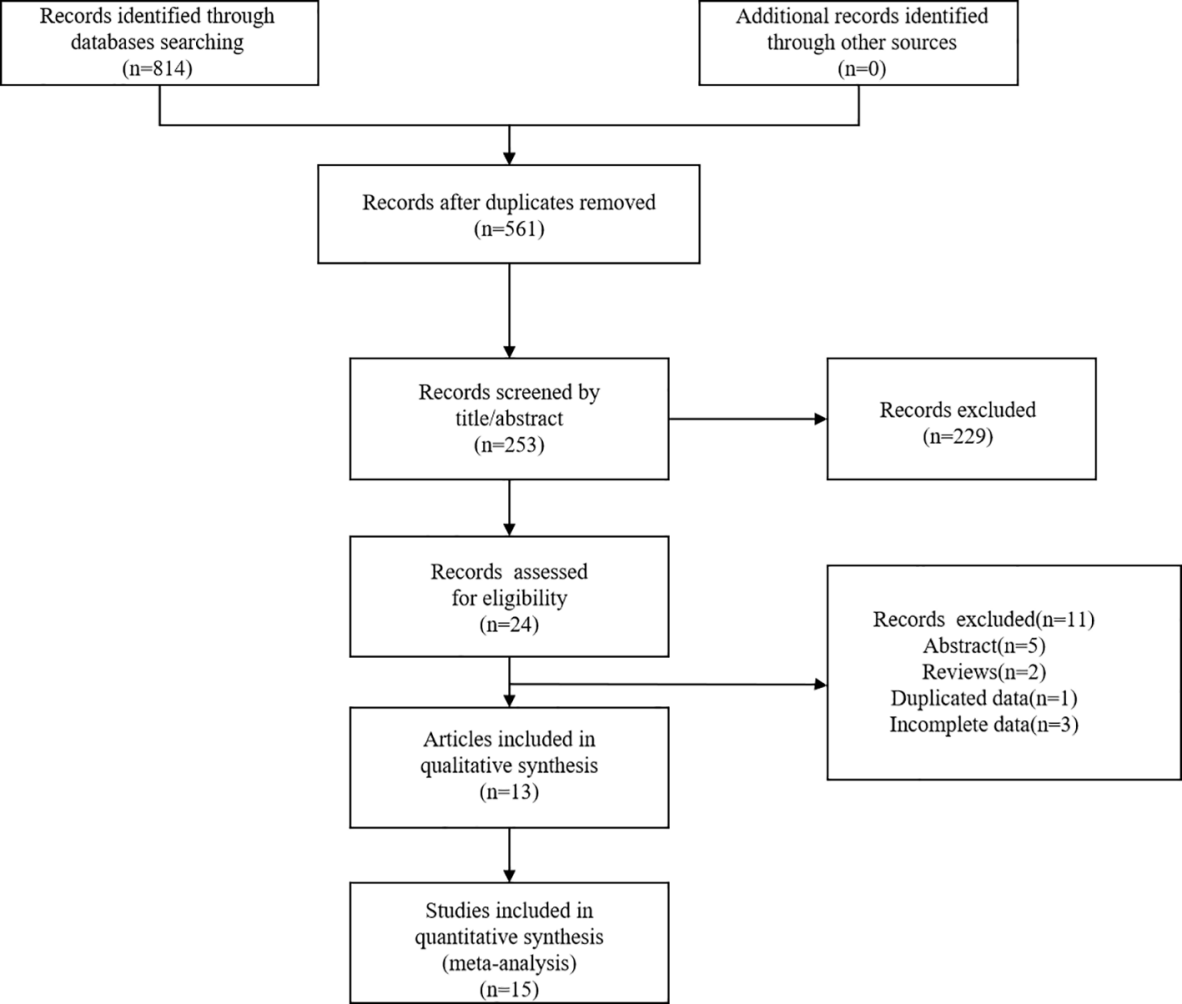
The flow diagram of identifying eligible studies.

A total of 814 articles were obtained through a preliminary query of relevant databases. After the removal of 253 duplicates, a total of 561 publications were further evaluated. After a review of titles and abstracts, 229 articles were further removed. Finally, 15 studies from 13 articles with a total of 1066 patients were included in the meta-analysis (22, 23, 28–38). **Figure 1** showed the literature search process.

### Study characteristics

**Table 1** summarized the main features of the included studies. 14 studies reported OS data and 4 studies analyzed DFS/PFS/RFS data. A total of four different tumors were reported, including prostate cancer, renal cancer, bladder cancer and upper tract urothelial carcinoma. NOS scores for the included studies ranged from 6 to 7, indicating a high quality of each study (**Supplementary Table S1**).

**Table 1:** Basic information of the included studies.

Study	Year	design	Sample size	Cancer type	Treatment methods	Analysis type	Survival analysis	NOS score
Bailey-Whyte	2023	R	680	PC	Non-surgery	MV	OS	7
Chen	2019A	R	414	RC	Surgery	MV	OS	7
Chen	2019B	R	168	RC	Surgery	UV	OS	6
Fukuda	2018	R	152	RC	Surgery	UV	OS	6
Gu	2017	R	161	RC	Surgery	MV	OS	7
Kadono	2021	R	91	BC	Non-surgery	UV	OS	7
Lv	2022	R	144	RC	Surgery	MV	OS, PFS	7
Mao	2021	R	343	RC	Surgery	MV	OS	7
Ni	2021	R	203	BC	Surgery	MV	OS, DFS	7
Tang	2023	R	820	RC	Surgery	MV	OS	7
Yilmaz	2022	R	241	BC	Surgery	MV	OS, RFS	7
Zapaia	2022	R	495	RC	Surgery	MV	OS	7
Zheng	2014A	R	259	UTUC	Surgery	MV	OS	7
Zheng	2014B	R	274	UTUC	Surgery	MV	OS	7
Ye	2022	R	540	BC	Non-surgery	UV	RFS, PFS	6

PC, Prostate Cancer; RC, renal cancer; BC, bladder cancer; UTUC, Upper Tract Urothelial Carcinoma; R, retrospective; OS, overall survival; DFS, disease-free survival; RFS, recurrence free survival; PFS, progression-free survival; MVA: multivariate analysis; UVA: univariate analysis; NOS score, Newcastle-Ottawa Scale score.

### Association between high SIRI and OS

**Figure 2:**
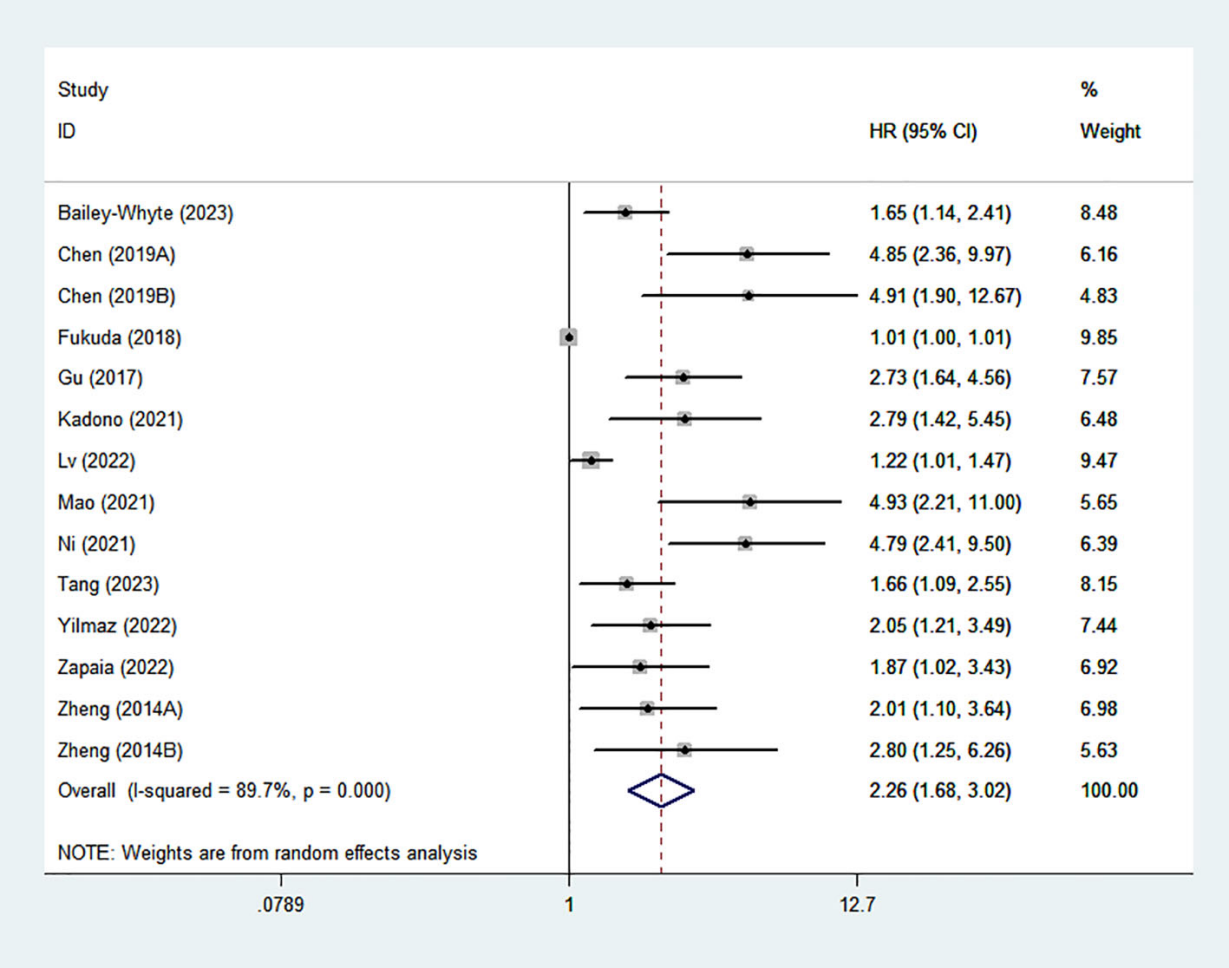
Forest plots of the relationship between SIRI and OS. SIRI, systemic inflammation response index; OS, overall survival;.

Fourteen studies evaluated the correlation between elevated SIRI and OS. Given the significant heterogeneity (I²=89.6%), a random-effects model was applied. The pooled HR was 2.16 (95% CI: 1.61–2.89), indicating that UM patients with elevated SIRI s had poorer OS. The result was shown in **Figure 2**.

### Subgroup analysis for OS

Subgroup analysis was further performed based on country, cancer type, treatment method and region (**Table 2**). The results showed that high SIRI predicted poor prognosis in both the surgical treatment group (HR:1.58; 95% CI:1.17-2.15) and the non-surgical treatment group (HR:2.02; 95% CI:3.00). Subgroup analysis also indicated that elevated SIRI mainly served as a poor prognostic marker in renal cell carcinoma, bladder cancer and upper tract urothelial carcinoma. In addition, subgroup analysis also showed that treatment method and cancer type may be the important source of heterogeneity.

**Table 2:** Subgroup analysis for OS.

Variables	No of studies	Estimate HR (95%)	P value	Heterogeneity I2 (%)	*p* value
Treatment					
Surgery	12	2.29(1.67-3.16)	<0.01	90	<0.01
Non-surgery	2	1.87(1.36-2.59)	<0.01	44	0.182
Country					
China	9	2.77(1.81-4.24)	<0.01	82.6	<0.01
Turkey	1	2.05(1.21-3.49)			
USA	1	1.65(1.14-2.41)			
Japan	2	1.58(0.586-4.261)	0.366	88.7	0.003
Poland	1	1.87(1.02-3.43)			
Region					
Asia	12	2.38(1.71-3.30)	<0.01	90.4	<0.01
Europe and America	2	1.71(1.24-2.35)	0.001	0	0.731
Analysis type					
UV	3	2.22(0.81-6.07)	0.122	89.9	<0.01
MV	11	2.31(1.71-3.13)	<0.01	76.1	<0.01
Cancer type					
PC	1	1.65(1.14-2.41)			
RC	8	2.09(1.47-2.98)	<0.01	90.3	<0.01
BC	3	2.81(1.97-4.01)	<0.01	45.7	0.158
UTUC	2	2.20(1.40-3.65)	0.001	0	0.518

PC, Prostate Cancer; RC, renal cancer; BC, bladder cancer; UTUC, Upper Tract Urothelial Carcinoma; MVA, multivariate analysis; UVA, univariate analysis.

### Association between high SIRI and DFS/PFS/RFS

**Figure 3:**
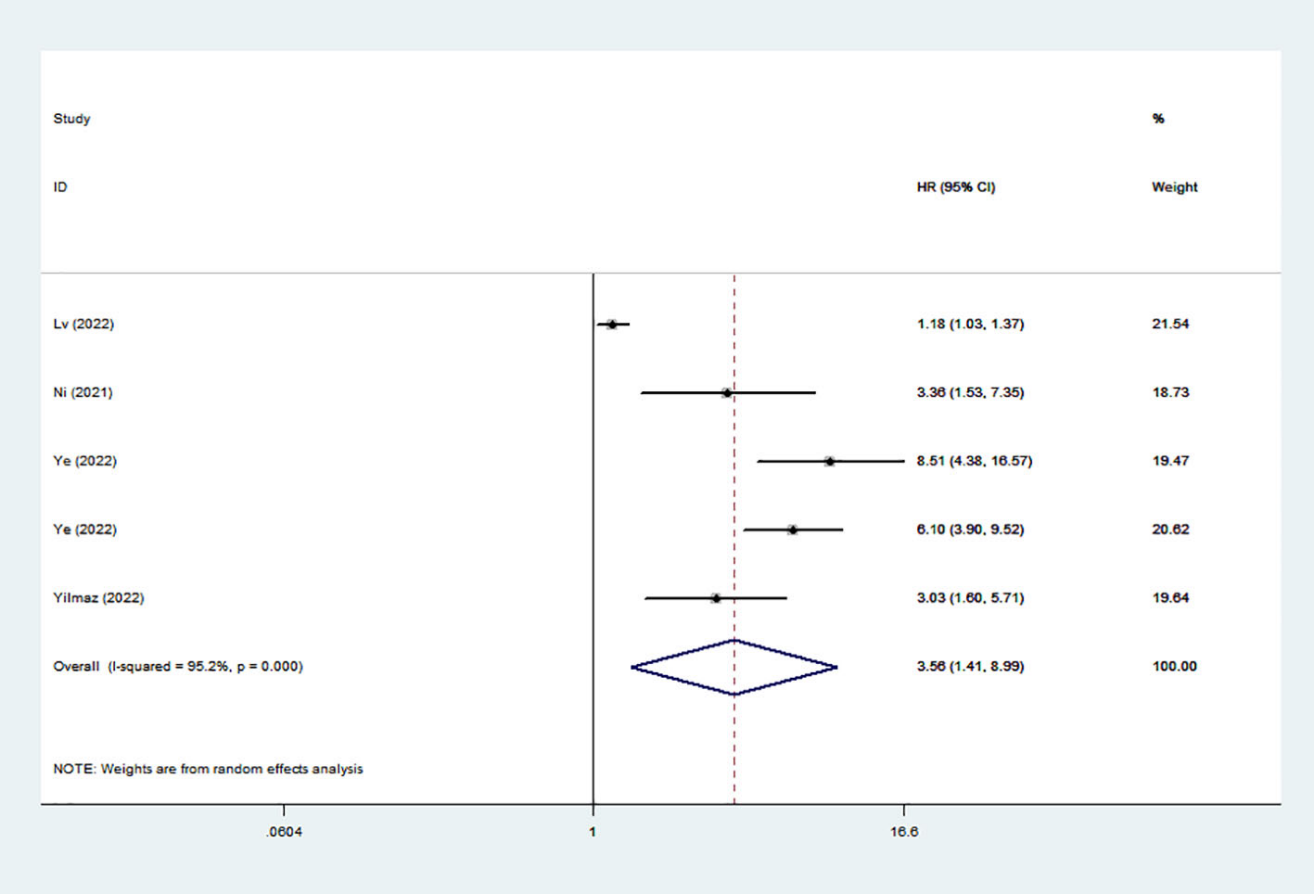
Forest plots of the relationship between SIRI and DFS/PFS/RFS. SIRI, systemic inflammation response index; DFS/PFS/RFS, disease-free survival/progression-free survival/recurrence-free survival.

Five studies reveled the relationship between SIRI and DFS/PFS/RFS. Owing to considerable diversity (I^2^ = 95.2%), a random-effects model was utilized. Our findings revealed a correlation between elevated SIRI and adverse DFS/PFS/RFS (HR: 3.56; 95% CI: 1.41–8.99) (**Figure 3**).

### Sensitivity analysis

**Figure 4:**
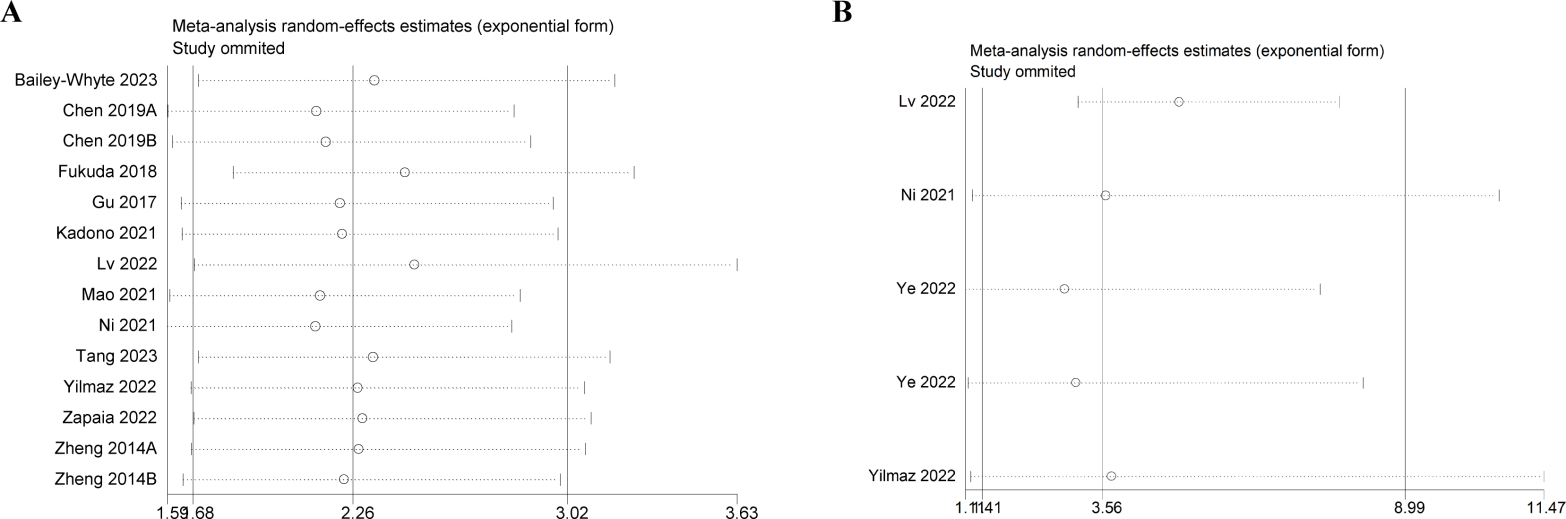
Sensitivity analysis. **(A)** Sensitivity analysis for OS. **(B)** Sensitivity analysis for DFS/PFS/RFS. OS, overall survival; DFS/PFS/RFS, disease-free survival/progression-free survival/recurrence-free survival.

Sensitivity analysis was performed by sequentially deleting one study. The results showed that no study had significant influence on the results of comprehensive analysis, indicating that the results of meta-analysis were stable and reliable (**Figure 4A, B**).

### Publication bias

**Figure 5:**
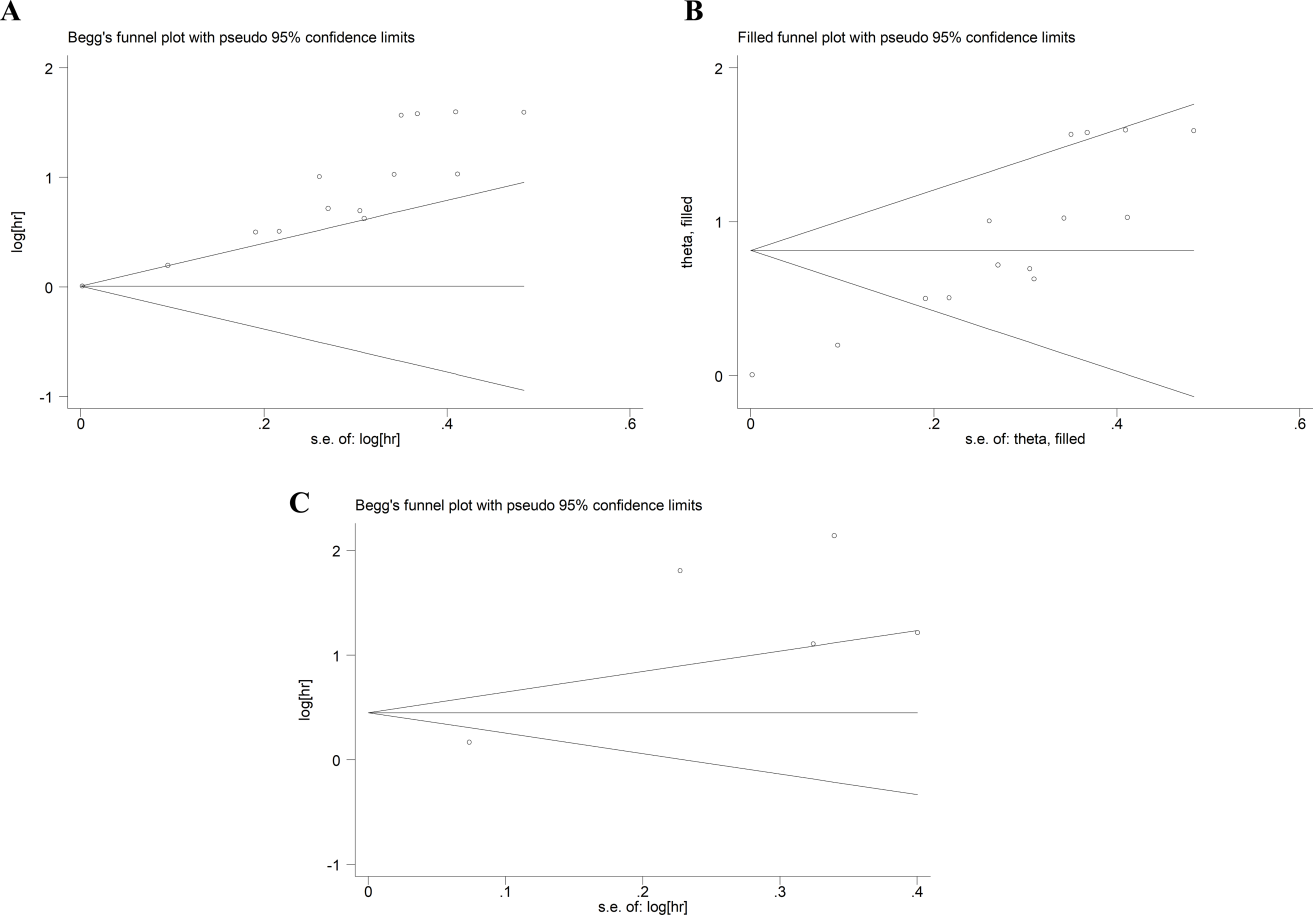
Publication bias. **(A)** Publication bias for OS. **(B)** The trim-and-fill method tested the OS data. **(C)** Publication bias for DFS/PFS/RFS. OS, overall survival; DFS/PFS/RFS, disease-free survival/progression-free survival/recurrence-free survival.

The evaluation of publication bias was performed using Begg’s test and Egger’s test. The *p*-values of Begg’s test and Egger’s test for OS were 0.059 and 0, respectively (**Figure 5A**). There was some publication bias for OS. However, trim-and-fill methods proved that the result was not affected by publication bias (HR: 2.26, 95% CI: 1.68–3.02) (**Figure 5B**). The *p*-values of Begg’s test and Egger’s test for DFS/PFS/RFS were 1 and 0.051, respectively (**Figure 5C**), suggesting there was no publication bias.

## Discussion

No studies have fully evaluated the prognostic significance of SIRI in patients with UM. 15 studies involving 4985 patients were included in the meta-analysis. The results showed that high SIRI was associated with adverse OS and DFS/PFS/RFS in patients with UM. Subgroup analysis showed that the prognostic value of SIRI in UM was not affected by treatment method. In addition, subgroup analysis also revealed that the prognostic value of SIRI was generalizable regardless of Asian or European-American populations. Furthermore, SIRI demonstrated better prognostic value in renal cell carcinoma, bladder cancer and upper tract urothelial carcinoma.

Increasing evidenced suggested that systemic inflammation significantly influences cancer growth, recurrence and progression, thereby affecting patient survival (6, 39). The systemic inflammation may precede malignant transformation, implying that an inflammatory microenvironment could facilitate tumor development (40). In addition, the emergence and progression of cancers were linked to interactions within the immune system (41). Research displayed obvious correlation between changes in regulatory T cells and tumor-related macrophages in non-muscle-invasive bladder cancer (NMIBC) patients and poor survival outcomes (42). Additionally, intravesical bacillus calmette-guerin immunotherapy increased the CD4+ T cell population more effectively than intravesical chemotherapy (43).Studies showed that viruses, especially HPV, may be a risk factor for urothelial carcinoma of the bladder (44–46). HPV infection caused inflammation and immune disorders in patients, thus promoting the occurrence of tumors (44).Targeting the progression of cancer by modulating specific inflammatory cytokines or immune cell has emerged as a promising therapeutic approach.

SIRI effectively predicted prognosis by evaluating the inflammatory and immune status of cancer patients. However, the specific mechanism that why SIRI determined the prognosis of cancer patients was unclear. We explained this phenomenon by analyzing SIRI composition parameters.

The critical role of neutrophils within the tumor’s immune microenvironment has garnered significant interest (47). Inflammation is essential for triggering tumor development through the damage of healthy tissues, and neutrophils played the crucial role in the process (48). Neutrophils entered different organs via CXCR2 ligands and performed immunosuppressive functions in the tumor microenvironment (49, 50). The reactive oxygen species and angiogenic factors produced by neutrophils could affect tumor initiation, progression and metastasis (51, 52). In addition, neutrophils can also promote the proliferation and differentiation of tumor cells by inhibiting lymphocyt-mediated cytolysis (53). Many studies confirmed that high blood neutrophils were closely related to poor prognosis of tumor patients (54).

At various stages of tumor development, monocytes are attracted by inflammatory mediators into the tumor microenvironment to exert specific immune functions (55). Studies showed that monocytes can differentiate into tumor-associated macrophages, which could degrade the extracellular matrix, induce immunosuppression, tumor angiogenesis and increase the likelihood of tumor metastasis (56). Data suggested that the prognosis of tumor patients with increased blood monocytes was generally adverse (57).

As an important part of immune system, lymphocytes act as an important role in immune defense. Lymphocytes could inhibit tumor progression by directly inhibiting tumor cell proliferation (58). Lymphocytes could activate cell-mediated immune responses and stimulate cytokine release to promote tumor lysis (59). However, a decrease in the numbers of lymphocytes can lead to immune dysfunction and immune dysfunction and immunosuppression. Evidence revealed that lymphocytopenia predicted significantly lower survival time in a variety of tumors, including UM (6, 60, 61).

High SIRI indicated high neutrophils or monocytes count and low lymphocytes counts, which reflected significant systemic inflammation and immunosuppression in tumor patients. Therefore, it was not difficult to understand that why high SIRI was associated with poor prognosis in patients with urinary tract tumors.

There were some defects in the study. Firstly, all included articles were retrospective studies. Secondly, most of the included studies were from China. Therefore, more large-scale studies from different regions were needed to further evaluate the prognostic value of SIRI in UM patients. Thirdly, Some urinary tumors, such as testicular cancer, was not evaluated. Fourth, our study was the grouping of different tumor types into a single category of urological malignancies. These cancers had distinct biological behaviors and prognostic factors, which may contribute to the observed heterogeneity (62–64). Finally, we were unable to assess the association between SIRI and clinicopathological features due to lack of data.

Our study also had some strengths. Firstly, this was the first meta-analysis to assess the prognostic value of SIRI in UM. Secondly, the sensitivity analysis confirmed that the results of the meta-analysis were stable. Thirdly, subgroup analysis further proved the prognostic value of SIRI. Fourth, the results of OS were not affected by publication bias through trim-and-fill method.

## Conclusions

Our study suggested that elevated SIRI was associated with poorer survival outcomes in UM patients. SIRI acts as an accessible serum indicator, offering a dynamic assessment of prognosis and treatment outcomes in UM patients. Clinicians can use SIRI to quickly assess UM patient’s nutritional status, immune function, and prognosis, providing guidance for individualized treatment. However, due to the limitations, further prospective studies were needed to validate our results.

## Data Availability

The original contributions presented in the study are included in the article/**Supplementary Material**. Further inquiries can be directed to the corresponding author/s.
