# Supplementary material for: Significant association between systemic inflammation response index and prognosis in patients with urological malignancies
Source: Front Immunol. 2025 Feb 26;16:1518647. doi: 10.3389/fimmu.2025.1518647 (PMC11897710; doi:10.3389/fimmu.2025.1518647)
Supplement: Supplementary file 1 [file Table1.docx]

| First author | Year | Selecion | Comparability | Outcome | Total |
| --- | --- | --- | --- | --- | --- |
| Bailey-Whyte | 2023 | ★★★ | ★ | ★★★ | 7 |
| Chen | 2019A | ★★★ | ★ | ★★★ | 7 |
| Chen | 2019B | ★★★ | ★ | ★★ | 6 |
| Fukuda | 2017 | ★★★ | ★ | ★★ | 6 |
| Gu | 2018 | ★★ | ★★ | ★★★ | 7 |
| Kadono | 2021 | ★★★ | ★★ | ★★ | 7 |
| Lv | 2022 | ★★ | ★★ | ★★★ | 7 |
| Mao | 2021 | ★★ | ★★ | ★★★ | 7 |
| Ni | 2021 | ★★ | ★★ | ★★★ | 7 |
| Tang | 2023 | ★★ | ★★ | ★★★ | 7 |
| Yilmaz | 2022 | ★★ | ★★ | ★★★ | 7 |
| Zapała | 2022 | ★★ | ★★ | ★★★ | 7 |
| Zheng | 2014A | ★★ | ★★ | ★★★ | 7 |
| Zheng | 2014B | ★★ | ★★ | ★★★ | 7 |
| Ye | 2022 | ★★ | ★★ | ★★ | 6 |

**Supplement table1.** Newcastle-Ottawa quality assessment scale.
